# Immunoglobulin G1 Allotype Influences Antibody Subclass Distribution in Response to HIV gp140 Vaccination

**DOI:** 10.3389/fimmu.2017.01883

**Published:** 2017-12-20

**Authors:** Sven Kratochvil, Paul F. McKay, Amy W. Chung, Stephen J. Kent, Jill Gilmour, Robin J. Shattock

**Affiliations:** ^1^Imperial College London, Medicine, London, United Kingdom; ^2^Department of Microbiology and Immunology, Peter Doherty Institute for Infection and Immunity, University of Melbourne, Melbourne, VIC, Australia; ^3^ARC Centre of Excellence in Convergent Bio-Nano Science and Technology, University of Melbourne, Melbourne, VIC, Australia; ^4^Melbourne Sexual Health Centre, Department of Infectious Diseases, Alfred Health, Central Clinical School, Monash University, Melbourne, VIC, Australia; ^5^IAVI Human Immunology Laboratory, Imperial College London, London, United Kingdom

**Keywords:** allotype, G1m3, G1m1, HIV, vaccines

## Abstract

Antibody subclasses exhibit extensive polymorphisms (allotypes) that could potentially impact the quality of HIV-vaccine induced B cell responses. Allotypes of immunoglobulin (Ig) G1, the most abundant serum antibody, have been shown to display altered functional properties in regard to serum half-life, Fc-receptor binding and FcRn-mediated mucosal transcytosis. To investigate the potential link between allotypic IgG1-variants and vaccine-generated humoral responses in a cohort of 14 HIV vaccine recipients, we developed a novel protocol for rapid IgG1-allotyping. We combined PCR and ELISA assays in a dual approach to determine the IgG1 allotype identity (G1m3 and/or G1m1) of trial participants, using human plasma and RNA isolated from PBMC. The IgG1-allotype distribution of our participants mirrored previously reported results for caucasoid populations. We observed elevated levels of HIV gp140-specific IgG1 and decreased IgG2 levels associated with the G1m1-allele, in contrast to G1m3 carriers. These data suggest that vaccinees homozygous for G1m1 are predisposed to develop elevated Ag-specific IgG1:IgG2 ratios compared to G1m3-carriers. This elevated IgG1:IgG2 ratio was further associated with higher FcγR-dimer engagement, a surrogate for potential antibody-dependent cellular cytotoxicity (ADCC) and antibody-dependent cellular phagocytosis (ADCP) function. Although preliminary, these results suggest that IgG1 allotype may have a significant impact on IgG subclass distribution in response to vaccination and associated Fc-mediated effector functions. These results have important implications for ongoing HIV vaccine efficacy studies predicated on engagement of FcγR-mediated cellular functions including ADCC and ADCP, and warrant further investigation. Our novel allotyping protocol provides new tools to determine the potential impact of IgG1 allotypes on vaccine efficacy.

## Introduction

Antibodies are generally accepted to contribute to vaccine induced protection against many infectious diseases including HIV (1). However, the involvement of different antibody classes or subclasses is less clear, where their different structural properties affect functional immunity. While antibody binding fragments (Fabs) are critical to determining binding specificity and neutralization, the Fc domain is the primary determinant for a wide spectrum of immunological functions mediated by the engagement of Fc-gamma receptors (FcγR) on a range of effector cells. These functions are regulated during an immune response through Immunoglobulin (Ig) subclass composition tailoring the selective interaction with FcγR on effector immune populations. Indeed, it has been suggested that Ig subclass composition may influence a wide range of Fc-mediated effector functions including antibody-dependent cellular cytotoxicity (ADCC) and antibody-dependent cellular phagocytosis (ADCP) (2). In this respect Fc-mediated effector functions likely augment the potency of broadly neutralizing antibodies (3) and are critical to the function of non-neutralizing antibodies. The observed modest efficacy of the RV144 HIV vaccine trial showing vaccine elicited protection in the absence of neutralizing antibodies (4) has driven an intense interest in the role of FcγR effector functions in protection and control of HIV infection (5, 6). Here the antibody subclass distribution is likely to play a critical role where IgG1/IgG3 interact efficiently with most FcγR, while IgG2/IgG4 show reduced affinity for many FcγR. Indeed, divergent antibody subclass profiles have been associated with variable antibody effector functions among HIV-1 controllers, where levels of HIV-specific IgG1/3 were the major distinguishing factor (7), while IgG2/IgG4 HIV specific antibodies were associated with poorer overall antibody activity (8). In this respect, inter-individual variation in the antibody subclass response profiles to HIV-1 infection and/or vaccination provides significant challenges in the development of a globally effective vaccine.

In addition to sequence diversity of variable Fab domains and isotypic variation, IgG-subclass antibodies have been shown to exhibit polymorphic epitopes (IgG-allotypes), which can differ between individuals and ethnic groups (2). Inherited in a codominant Mendelian fashion, IgG-heavy chain allotypes are designated as natural genetic marker (Gm) together with the antibody subclass (e.g., G1m) and the allotype number (e.g., G1m3 or G1m1) (9). So far, a total of 4 G1m human allotypes: G1m17, G1m3, G1m1, and G1m2; two G1m alloallotypes: G1m27 and G1m28; and two G1m isoallotypes: nG1m17 and nG1m1 have been identified *via* serological typing (10). These define 7 G1m alleles: G1m17,1; G1m3; G1m17,1,27; G1m17,1,28; G1m17,1,27,28; G1m17,1,2; and G1m3,1; where the G1m1 allotype is common to all alleles except G1m3. The prevalence of these alleles broadly differs according to European, African or Asian ancestry. Most Gm allotypes are located in the Fc-region (CH2 or CH3) of antibodies, with the exception of G1m3 which is linked to amino acid changes in the CH1-region: expressing Arg rather than Lys at position 120 (2, 10). G1m3 also expresses unique amino acids at positions 356 (Glu) and 358 (Met) in CH3 as opposed to Asp/Leu common to all G1m1 allotypes. While allotypes are encoded by one given Ig gene, some amino acid variations can be found in antibody chains of other isotypes (isoallotypes). For example, the amino acid residue Arg120, which corresponds to G1m3, is also found in antibodies belonging to the IGHG3 and IGHG4 allele family (10, 11).

Prior work has linked Gm allotypes in the Ig constant heavy G chain (IGHG) to augmented antibody responses against certain diseases (12–15). For example, IgG antibody responses against the hepatitis C virus envelope proteins E1E2 in a cohort of infected subjects with GM 1,17 5,13 and KM 1 phenotypes exhibit fourfold higher levels of E1E2-specific antibodies (13). Another study showed that IgG1 antibody levels to malaria vaccine-antigens were significantly higher in subjects with the GM 3 23 5,13,14 phenotype when compared to subjects lacking this phenotype (16).

Similar trends have been reported for IgG-subclass and specificity profiles in a cohort of elite HIV-1 controllers where HIV-specific IgG1 levels correlated with Fc-dependent effector functions and total plasma IgG1. Subsequently, it was argued that Gm allotypes could be responsible for variations in IgG1-concentrations and it was suggested that future studies should incorporate Gm allotyping protocols to account for this possibility (7). Furthermore, previous studies have demonstrated the involvement of Gm alleles in ADCC of cancer cells (17, 18). Taken together these studies indicate that Gm allotypes impact on antibody functionality (19) and provided a strong rationale for investigating the impact of IgG1-allotypes on the magnitude and functionality of vaccine-induced IgG-subclasses responses in the context of an HIV-vaccine trial.

Traditionally Gm phenotype has been determined *via* hemagglutination inhibition assays (HAI) using anti-Rh IgG antibodies of known allotypy, and polyclonal IgG of a selected allotype-specificity (e.g., antihuman G1m3). However, access to such reagents can be rate limiting and the approach is less amenable to high volume screening. Given the dominant role of IgG1 responses to HIV-1 envelope immunogens (20), we sought to determine the impact of IgG1-allotypy on the magnitude of induced responses. Here, we combined PCR and ELISA assays in a dual approach to determine the IgG1 allotype identity (G1m3 and/or G1m1) of clinical trial participants, using human plasma and RNA isolated from PBMC. Subsequently, the distribution of IgG1-allotypes formed the framework for assessing the effect of IgG1-allotypes on the magnitude and functionality of vaccine-induced antibody responses. Understanding how IgG1 allotype influences IgG subclass distribution in response to vaccination may prove an important consideration in the design and evaluation of vaccines strategies across ethnic groups.

## Materials and Methods

### HIV Vaccine Trials

This study mainly builds upon findings from the previously published X001 clinical trial (21), registered at http://ClinicalTrials.gov under no. NCT01966900, EudraCT 2013-001032-22. In brief, a recombinant clade C HIV-1 envelope gp140 protein (CN54gp140) produced by Polymun Scientific (Klosterneuburg, Austria) to GMP specification, which has been reported to be immunogenic in a number of preclinical and clinical studies (22, 23), was used. The vaccine antigen CN54gp140 was administered intramuscularly into the deltoid muscle of the upper arm at a dosage of 100 µg CN54gp140 formulated with 5 µg GLA-AF [Glucopyranosyl Lipid A—Aqueous Formulation, Infectious Disease Research Institute, Seattle, USA (24)] in a total volume of 0.4 ml at weeks 0, 4, and 8 with a boost inoculation with the same material at either month 6 or 12. The trial was performed at the NIHR/Wellcome Trust Imperial Clinical Research Facility, Imperial College, London. The trial population was predominantly Caucasian, although subject ethnicity was not recorded as part of the trial. Samples were also obtained from the MUCOVAC2 study (23) registered with the UK Clinical Research Network (UKCRN) Number 11679, EudraCT 2010-019103-27. In brief, recombinant CN54gp140 was administered by intramuscular (IM), intranasal (IN), or intravaginal routes of administration in HIV negative female volunteers. Sera was obtained for this study from subjects receiving IM immunizations administered at the same dosage as the X001 trial (100 μg with 5 μg GLA-AF) with the same schedule (0, 4, and 8 weeks). The trial population was also predominantly Caucasian.

### Ethics Statement

The clinical trials generating serum and PBMC samples were conducted in compliance with UK Clinical Trial Regulations and any amendments, which include compliance with the principles of Good Clinical Practice, and the study abided by the principles of the Declaration of Helsinki. All volunteers provided written informed consent to participate in the trials on the basis of appropriate information and with adequate time to consider the information and discuss the trial with the Principal Investigators or their delegate. The trial proposal, the trial-specific information provided to volunteers, the consent form and substantial protocol amendments (if applicable) were reviewed by a recognized Research Ethics Committee and by the Medicines and Healthcare products Regulatory Authority (see EudraCT numbers above). All volunteers were made aware that they were free to withdraw without obligation at any time and that such an action would not adversely affect any aspect of their medical care or legal rights.

### PBMC Isolation

PBMC were isolated using density gradient separation from heparinized whole blood, used fresh (within 8 h of blood collection) or frozen in a mixture of fetal bovine serum (Sigma-Aldrich, St. Louis, MO, USA) and DMSO at a 90:10 ratio using a Kryo 560-16 rate controlled freezer (Planer, Sunbury-On-Thames, UK). PBMC were stored in vapor phase liquid nitrogen.

### Determining the IgG1-allotype Identity

Human RNA was isolated from PBMC with the RNeasy Mini Kit for RNA (Qiagen, UK) and transcribed into cDNA *via* oligo(dT)_18_ primers, using the maxima first strand cDNA synthesis kit (Cat: K1672, Thermo Scientific, UK). Subsequently, the template cDNA was used for a primary PCR for both G1m3 and G1m1 allotyping. While the primary PCR is sufficient to account for G1m1,17 alleles, G1m3-allotyping required a secondary PCR to take account of (and exclude) isoallotypes that can be present in IgG3 and IgG4 regions. Primers and PCR programs are detailed in the supplementary section (Tables S1–S5 in Supplementary Material).

### Agarose Gels

DNA products were separated by 1.2% agarose gel electrophoresis (100 V, 1 h) in 1× Tris acetate EDTA buffer. The agarose gel was stained with SYBR Safe (1 in 20,000), a suitable DNA ladder was loaded and 5× loading dye was added to each sample prior to loading the gel. DNA was visualized on a transilluminator.

### ELISA Protocol for IgG1-Allotyping

#### G1m3-ELISA

Serum antibodies from clinical trial participants were assessed for the presence of the IgG1-allotype G1m3 *via* a novel ELISA protocol adapted from a previously published ELISA platform (21). This antibody recognizes both the G1m3 allele prevalent in Caucasian populations and the G1m1,3 allele prevalent in those with Asian ancestry. In brief, 96-well high binding MaxiSorp plates (Nunc) were coated with 100 μl/well anti-G1m3 (Cat: I5385-0.2ML, Sigma, UK), at a 1:5,000 dilution in PBS, overnight at 4°C. As reference material, standard human Igs, which were captured with a combination of anti-human kappa (Southern Biotech, Cat: 2060-01) and lambda light chain (Cat: 2070-01) specific mouse antibodies, were used. These capture antibodies were coated onto the plates overnight at 4°C and coated plates were washed four times with PBS-T before blocking with PBS supplemented with 1%BSA and 0.05% Tween-20. Following further washing, diluted serum samples were added to the precoated wells (generally between 1:10,000 and 1,000,000) and titrations of Ig standards were added to the kappa/lambda capture antibody coated wells at 50 μl/well and incubated for 1 h at 37°C. Plates were washed four times prior to the addition of antihuman IgG-HRP and incubated for 1 h at 37°C. Plates were washed four times and developed with 50 μl/well of KPL SureBlue TMB substrate (Insight Biotechnology, UK). The reaction was stopped after 5 min by adding 50 μl/well of 1 M H_2_SO_4_, and the absorbance read at 450 nm on a KC4 spectrophotometer.

#### G1m1 ELISA

For the G1m1 ELISA the commercially available detection antibody anti-IgG1-Hinge-HRP (Cat: 9052-08, Southern Biotech) was used at a 1:5,000 dilution. Except for the detection antibody, the G1m1-ELISA is identical to the anti-IgG1-ELISA protocol previously published (21). This antibody does not bind to G1m3 allele, but does show cross reactivity with G1m1,3 allele prevalent in those with Asian ancestry, suggestive of recognition of the common G1m1 allotype.

### Customized Multiplex Dimer Assay for the Assessment of FcR-Binding

A customized multivariate multiplex assay was developed using a panel of gp140 antigens (Clade C: CN54, 1086, Clade A: UG37, Clade D: UG21–NIH AIDS Reagents) covalently conjugated to different magnetic fluorescent multiplex beads (Bio-Rad, AU) as described previously (25). Biotinylated dimeric Fc-gamma-Receptors (FcγRIIa-H131, FcγRIIIa-V158) were produced as previously described (26). The dimeric FcγR multiplex method has been previously published (21). Briefly, gp140 coupled microspheres (minimum 500 of each individual antigen bead set per well) was added to 1:100 plasma diluted in PBS + beads, incubating overnight at 4°C. HIVIG was used as a positive control to normalize across multiple replicates. Beads were washed using a Bio-Rad magnetic plate-washer (Bio-Plex Pro Wash station) and incubated for 2 h with biotinylated dimeric FcγR (1.0 μg/ml), then subsequently washed and incubated 1 h with streptavidin PE (1.0 μg/ml), washed again before resuspending in sheath fluid. A Bio-plex MAGPIX and Bio-Plex Manager software (Bio-Rad) was used to detect the median fluorescence intensity (MFI) for each bead set.

### Statistical Methods

Immunological analyses were based on the per protocol population that received all vaccinations. Appropriate comparative statistics are annotated in the text and/or figure captions. Statistical analysis was carried out using Prism 7.0a (GraphPad, CA, USA) or the R software (R3.3.2) for statistical programming (27). The non-parametric Mann–Whitney test was used to compare two groups and the non-parametric Spearman’s rank correlation coefficient was used to interrogate correlative relationships between the distributions of HIV-specific IgG1 levels and Fc-receptor binding in X001 study participants. *p*-Values ≤0.05 were considered significant (*p ≤ 0.05, **p ≤ 0.01, and ***p ≤ 0.001).

## Results

### Cross-Validation of a Novel ELISA Protocol for Rapid IgG1-Allotyping against a PCR Protocol

**Figure 1:**
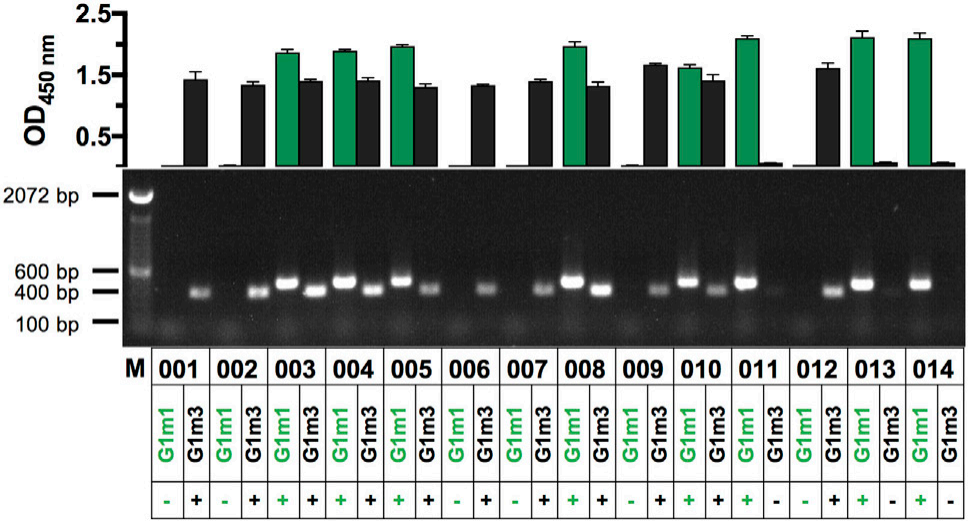
Assessment of IgG1-allotypes G1m3 and G1m1 in a cohort of HIV vaccine recipients *via* PCR and ELISA (*n* = 14). In the top chart OD-values for G1m3-specific signals (black bars) and G1m1-specific signals (green bars) are shown for X001 study participants (labeled 001–014). Shown are the mean (±SD) OD_450_-values of technical triplicates, representative of three replicates. OD-values greater than 0.5 were considered to be positive for the respective IgG1-allotype G1m3 and/or G1m1. The bottom panel shows the corresponding results from a novel PCR protocol, which was used to determine IgG1-allotypes from human RNA. Band sizes are 400 bp for G1m3 and 463 bp for G1m1 as indicated by the marker on the left.

We wished to assess whether IgG-allotypes might be linked to differences in the IgG-subclass profile of Ag-specific antibody responses generated in the context of HIV-1 vaccination (28, 29). To pursue this, we developed novel PCR (Figure S1 in Supplementary Material) and ELISA protocols and combined them in a dual approach to determine the IgG1 allotype identity (G1m3 and/or G1m1) of clinical trial participants, using both human serum and mRNA. The optimized protocol was applied to determine the IgG1 allotype identity of 14 clinical trial participants from an HIV vaccine study utilizing a Clade C gp140 envelope protein (Figure 1; Figure S2 in Supplementary Material) (21). IgG1-allotyping X001 study participants revealed that 3/14 (21%) were homozygous for G1m1 and 6/14 (43%) homozygous for G1m3, with 5/14 (36%) carrying both alleles (heterozygous). The IgG1 allotype abundance mirrors previously reported most frequent alleles representative of Caucasian population enrolled in this study (G1m3; G1m 17, 1; and G1m17,1,2), the concurring results demonstrate the interchangeability of the PCR and ELISA protocol for IgG1-allotyping. The clear cross-validation of both assays formats, allowed for the determination of the IgG1-allotype abundance in two additional clinical trials (23, 30), from which only serum samples were available (Figure S3 in Supplementary Material).

### Implications of IgG1-Allotypes for the Analysis of HIV-Vaccine Induce IgG-Subclass Responses

**Figure 2:**
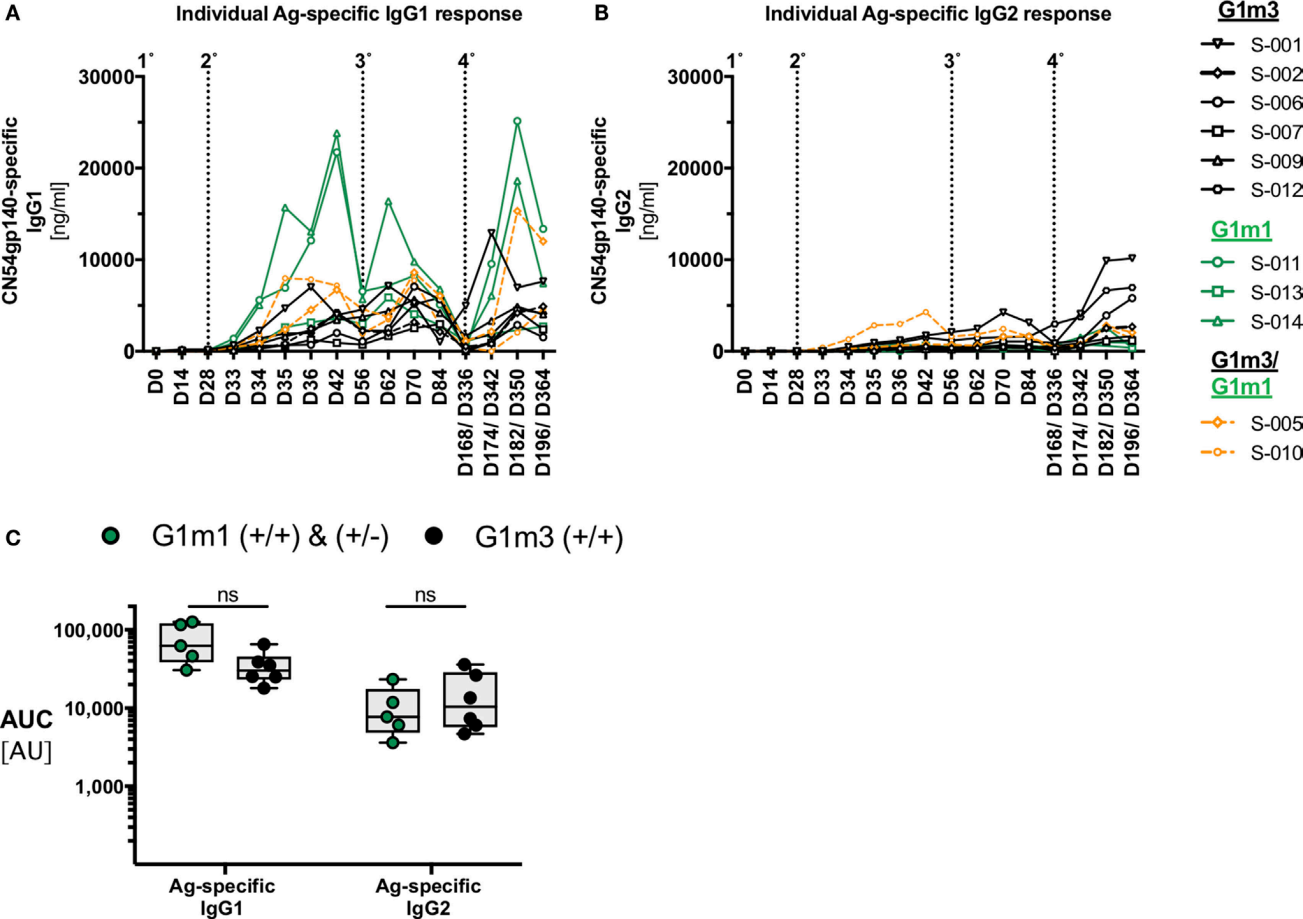
Individual antigen-specific IgG-subclass responses in the context of IgG1-allotypes. IgG-subclasses responses, classified with respect to G1m3 and/or G1m1 allotypes. **(A)** Individual Ag-specific IgG1-responses (participant IDs are shown in the legend on the right). **(B)** Individual Ag-specific IgG2-responses. **(C)** Area under curve (AUC) analysis for Ag-specific IgG1/IgG2 responses across all study time-points. Non-parametric Mann–Whitney test.

The IgG1-allotype identities determined for the X001 study (21) provided a distinctive framework for the re-analysis of HIV vaccine-induced IgG-subclass profiles of the 11 per protocol individuals included in the full immunological analysis (Figure 2). Most participants, homo- or heterozygous for G1m1, exhibited a trend for higher Ag-specific IgG1 concentrations when compared to homozygous G1m3-carriers (Figure 2A). There was also a potential trend for homozygous G1m3-carriers to have greater Ag-specific IgG2 responses following the fourth immunization (Figure 2B). By contrast there were no identified differences in Ag-specific IgG3 and IgG4 responses according to G1m1 allotype (data not shown). Although the X001 study (21) was not powered sufficiently to demonstrate a statistical significance between homologous G1m1- and G1m3-allele carriers, the apparent differences in Ag-specific IgG1/IgG2 levels warranted further investigation.

All participants with at least one G1m1-allele were then analyzed as a single group and directly compared with homozygous G1m3-carriers. The subsequent area under curve (AUC)-analysis across all time points helped to further unravel the effect of G1m1-allotypy on the magnitude of Ag-specific IgG1/IgG2 responses by revealing trends and potential differences between G1m1-carriers and homologous G1m3-carriers (*p* = 0.0823, Figure 2C). Furthermore, the highest Ag-specific IgG1 responses that occurred 14 days after the second IM and 14 days after the fourth IM were in G1m1-carriers (Figure 2A).

### Association of IgG1-Allotypes and Differences in Ratios of Antigen-Specific IgG1/IgG2-Levels following Serial Immunizations with CN54gp140

**Figure 3:**
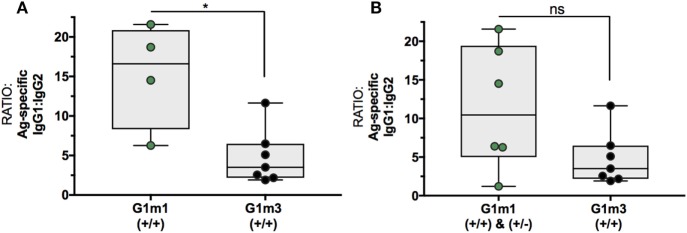
Differences in Ag-specific IgG1/IgG2 levels are associated with IgG1-allotypes after the third IM with CN54gp140. Presented data is from MUCOVAC2 and X001 trials and is dose/time matched. **(A)** Differences in Ag-specific IgG1/IgG2 ratios between homozygous G1m1- and G1m3-carriers (+/+). **(B)** Differences in Ag-specific IgG1/IgG2 ratios between homo-/heterozygous G1m1- and homozygous G1m3-carriers (+/+). Non-parametric Mann–Whitney test (*p < 0.05).

Following these initial observations, additional serum samples from the related MUCOVAC2 trial [EudraCT 2010-019103-27 (23)] were made available for IgG1-allotyping (Figure S2 in Supplementary Material). MUCOVAC2 is a predecessor study to X001 and was designed to establish the optimal route and dosage of immunization with the candidate HIV-1 clade C CN54gp140 envelope glycoprotein vaccine (23). The timing (third IM, week8), immunogen (CN54gp140), and dose (100 μg) in the MUCOVAC2 trial was identical to week 8 (third IM) in the X001 study. Thus, it was possible to pool data from the two clinical trials for this selected time point, allowing for a follow-up analysis of the differences in Ag-specific IgG1/IgG2 ratios mediated by G1m1 (Figure 3A: homozygous, *n* = 4; Figure 3B: homo- and/or heterozygous, *n* = 6) and G1m3 (homozygous, *n* = 7).

Following the 3-immunization priming phase, volunteers homozygous for G1m1 (*n* = 4) had fivefold higher Ag-specific IgG1/IgG2 ratios in comparison to homozygous G1m3-carriers (*n* = 7, *p* = 0.0242, Figure 3A). However, no significant differences in Ag-specific IgG1/IgG2 ratios were observed when comparing both hetero- and homozygous G1m1-carriers with homozygous G1m3-carriers when using this larger data set (*p* = 0.1807, Figure 3B). It is important to note that the difference in IgG1/IgG2 ratios when comparing homozygous G1m1 to G1m3 carriers likely reflects differences in magnitude of IgG1 responses, given there was little evidence for higher IgG2 responses at this time point (Figure 2B).

### Correlational Relationships between FcγR-Binding (FcγRIIa/FcγRIIIa) and Ag-Specific IgG1 Levels Were Determined with Respect to Different Combinations of G1m1 and/or G1m3 Alleles

**Figure 4:**
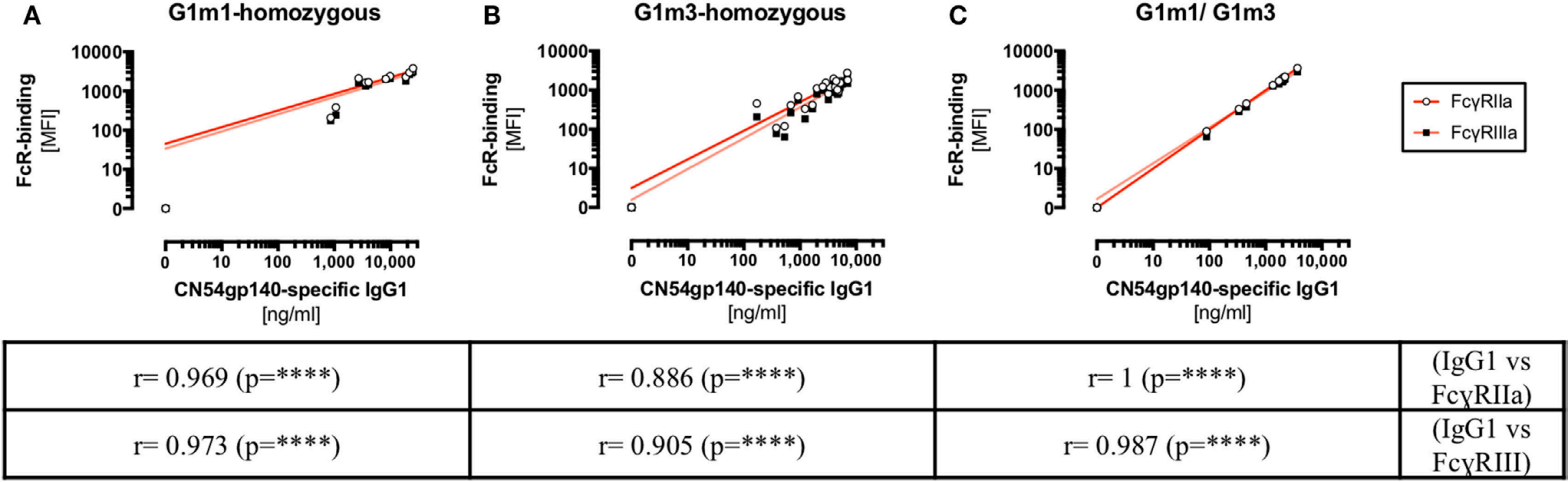
HIV-specific IgG1 levels (*x*-axis) correlate with Fc-receptor binding in X001 study participants. Correlation analysis for X001 study participants across all study time-points. **(A)** homozygous G1m1-carriers, **(B)** homozygous G1m3-carriers, and **(C)** individuals heterozygous for G1m1/G1m3. Below the graph Spearman’s rank correlation coefficient *r*-values are shown (****p < 0.0001). MFI, median fluorescence intensity.

Despite statistical limitations in study power, evidence was found to suggest that Ag-specific IgG1/IgG2 levels varied according to the IgG1-allotype of the HIV-vaccine recipients. To further elucidate the role of allotypic variations in antibody responses, the impact of IgG1-allotypes on the magnitude of Fc-mediated functions was investigated. Investigating these observations in the context of an HIV-vaccine trial was facilitated by the use of a novel assay, using FcγR-ectodomains for probing Fc-mediated functions (26, 31). This assay was chosen based on the ease of standardization across laboratories in comparison to the varied cellular models of ADCC and ADCP function. Correlational relationships between FcγR-binding (FcγRIIa/FcγRIIIa) and Ag-specific IgG1 levels were determined with respect to different combinations of G1m1 and/or G1m3 alleles in X001 study participants (Figure 4; Figure S4 in Supplementary Material). The engagement of FcγRIIa/FcγRIIIa dimers *via* CN54gp140-sepcifc serum antibodies correlated significantly (*p* < 0.0001) with Ag-specific IgG1 levels, irrespective of the IgG1-allotype combinations.

## Discussion

A novel allotyping protocol was developed and employed to determine the abundance of IgG1-allotypes in HIV vaccine studies (X001 and MUCOVAC1 clinical trials). The allotype abundance was found to mirror those previously reported for Caucasian populations, in which the G1m3-allele is known to be predominant (10, 15, 32, 33). Interestingly, the frequency of donors homozygous for G1m3 (45.4%) reported for a larger study (570 community blood donors) coincided with the G1m3-distribution determined for the X001 study (29). It is important to note that abundance of different G1m phenotypes and allotype differ in other ethnicities (e.g., Asian and African populations) for which many of the larger HIV vaccine trials are currently conducted, and these protocols will need to be confirmed in these ethnicities in the future.

Immunoglobulin G1 allotype analysis provided a framework for regrouping and reanalyzing the X001 Ag-specific IgG-subclass data. Initial data analysis of individual Ag-specific IgG1 responses in study participants suggested a link between G1m1-carriers (homozygous or heterozygous) and elevated Ag-specific IgG1 responses when compared to homozygous G1m3-carriers. Thus, Ag-specific IgG1 concentrations from both homo- and heterozygous G1m1 carriers were grouped and compared with homozygous G1m3-carriers, revealing differences in Ag-specific IgG1 levels between the two study groups. The results align well with a previous study, in which different lower serum IgG and IgG-subclass levels were associated with G1m3, G3m5 allotypes in a study population of 157 Caucasian blood donors (34) and mirror the observations made by Lai et al. (7).

In contrast to this finding, HIV-1 vaccine recipients with at least one G1m1 allele appeared to have lower Ag-specific IgG2 levels in comparison to homozygous G1m3-carriers following four immunizations. Subsequently, the Ag-specific IgG1:IgG2-ratio was compared for time- and dose-matched samples from two additional clinical HIV-vaccine trials. The data suggest that vaccinees homozygous for G1m1 have elevated Ag-specific IgG1:IgG2 ratios compared to G1m3-carriers. It is likely that this difference is predominantly driven by higher IgG1 levels associated with the G1m1 allele, any additional contribution of enhanced IgG2 levels associated with G1m3-carriers would need to be confirmed in larger studies. Interestingly, homozygosity for the G1m3-allele is strongly linked to the G2m23-allele in Northern Europe (9). Previous studies have suggested that individuals homozygous for this allotype (G2m23) exhibit higher serum IgG2 titers and decreased antipolysaccharide IgG2 responses than those homozygous for G2m(..), heterozygotes having intermediate levels (9, 12, 19, 35). Further work would be required to determine any additional contribution of G2m23 to the differences in IgG1:IgG2 ratios observed here.

Immunoglobulin G2 antibodies are generally associated with responses to carbohydrate antigens, which may be advantageous for recognizing ENV glycans (2), however, IgG2 has also been shown to recognize protein antigens. Indeed, two studies have demonstrated a link between IgG2 antibodies to HIV Gag proteins and natural control of HIV infection (36, 37). Nevertheless, a related study failed to detect differences in Gag-specific IgG2 levels between HIV controllers and chronic progressors (20). However, in contrast to the two studies, that used viral lysates in Western blot assays, Banerjee et al. (20) used recombinant HIV antigens in ELISAs, implying that conformational variations in HIV antigen could have affected IgG2-detection (20).

Observations in this study that elevated levels of HIV-specific IgG1 and decreased IgG2 levels were associated with the G1m17-allele, support the hypothesis that allotypes could present useful Gms for the assessment of HIV-1 acquisition risk in vaccinated individuals. This concept was first tested on samples from the Step Study (17), that failed to show protective efficacy. This phase IIb proof-of-concept study, designed to assess the efficacy of the MRK Ad5 gag/pol/nef HIV vaccine, was terminated prematurely on the grounds of futility. However, there was an observed increased risk of HIV-1 acquisition in the vaccine group when compared to the placebo group (38, 39). Pandey et al. (17) went on to investigate whether this observed increased risk of HIV-1 acquisition could be linked to Ig allotype and revealed that the risk of HIV-1 acquisition was significantly increased in individuals positive for a combination of homozygous G1m17 and Km1 allotypes. Furthermore, the researchers found that subjects homozygous for FcγRIIIa (F-version) in the absence of G2m23 were more likely to become infected with HIV-1 (17). The significance of these findings in terms of immune function are unclear given the vaccine approach was predicated on eliciting cellular responses designed to control viral replication rather than protective antibodies. Nevertheless, they serve to highlight the potential association of Ig allotype with HIV acquisition.

In the present study, we applied a novel assay for probing the engagement of FcγRIIa/FcγRIIIa dimers to investigate potential links between IgG1-allotypy and Fc-effector binding profiles (26). This has potential implications for HIV vaccine research since the significance of Fc-effector functions has been highlighted by a large phase III HIV-vaccine trial (RV144), in which HIV-specific ADCC was associated with enhanced protection against HIV-1 acquisition (40, 41). A similar vaccine approach is now being pursued in the HVTN 702 trail evaluated in South Africa. The potential for IgG1 allotype to influence ADCC function is not without precedent, previously reported in prostate cancer where the capacity of NK cells to mediate ADCC against prostate cancer cells is influenced by interactions between different IgG1-allotypes and the corresponding FcγRIIIa variants (28). Similar to the RV144, we determined the interactions of serum antibodies specific to different HIV-1 clades, with FcγRIIa/FcγRIIIa-dimers and with respect to the IgG1-allotypes G1m1 and G1m3. Higher titers of FcγR-dimer binding by CN54gp140-specific serum antibodies were detected in G1m1-carriers as opposed to homozygous G1m3 carriers. Increased IgG1-titers that were associated with G1m1 are likely responsible for augmented binding of FcγR dimers given the higher affinity of IgG1 over IgG2. Indeed, IgG1 titers directly correlated with FcγR-binding irrespective of allotype (Figure 4). This trend for increased FcγR-dimer binding in G1m1-carriers by vaccine-induced serum antibodies was generally preserved against envelope proteins of different HIV-1 clades, implying that epistatic interactions between Fc-domains and FcγR could play an important role in HIV-1 vaccines. This notion is supported by another study that investigated the abundance of FcγR and Gm allotypes among HIV-1 controllers and non-controllers. The major finding was that among Caucasian Americans negative for the FcγRIIa allele, Gm21-positive individuals were seven times more likely to be HIV-1 controllers than non-carriers of Gm21, whereas this trend was not observed in the African American cohort (42). It would be interesting to investigate at the sequence level, if similar links could be found for G1m3 and G1m1 allotypes in HIV-1 vaccine recipients.

Although the results of this study are preliminary, they suggest that individuals homozygous for G1m3 exhibit lower levels of Ag-specific IgG1 and as a consequence lower FcγR-engagement in response to HIV-vaccination. If FcγR function is important for antibody-mediated protection then these individuals would be less well protected than those homozygous for G1m1. These results have important implications for the two ongoing efficacy studies of HIV vaccines (HVTN 702 and 705) predicated on engagement of FcγR-mediated cellular functions including ADCC and ADCP. Our novel allotyping protocol provides new tools to determine the potential impact of IgG1 allotypes on vaccine efficacy.

## Ethics Statement

The clinical trials generating serum and PBMC samples were conducted in compliance with UK Clinical Trial Regulations and any amendments, which include compliance with the principles of Good Clinical Practice (GCP), and the study abided by the principles of the Declaration of Helsinki. All volunteers provided written informed consent to participate in the trials on the basis of appropriate information and with adequate time to consider the information and discuss the trial with the Principal Investigators or their delegate. The trial proposal, the trial-specific information provided to volunteers, the consent form and substantial protocol amendments (if applicable) were reviewed by a recognized Research Ethics Committee (REC) and by the UK Medicines and Healthcare products Regulatory Authority (MHRA). All volunteers were made aware that they were free to withdraw without obligation at any time and that such an action would not adversely affect any aspect of their medical care or legal rights.

## Author Contributions

RS, PK, and JG conceived the project. SK and PK designed and performed experiments, analyzed the data, and together with RS composed the manuscript. AC and SK designed and performed a customized Fc-dimer multiplex assay, and preprocessed the data, which was analyzed by SK and AC.

## Conflict of Interest Statement

The authors declare that the research was conducted in the absence of any commercial or financial relationships that could be construed as a potential conflict of interest. The handling editor declared a past coauthorship with the authors.
