# Corrigendum: Immunoglobulin G1 Allotype Influences Antibody Subclass Distribution in Response to HIV gp140 Vaccination

**DOI:** 10.3389/fimmu.2018.00342

**Published:** 2018-02-27

**Authors:** Sven Kratochvil, Paul F. McKay, Amy W. Chung, Stephen J. Kent, Jill Gilmour, Robin J. Shattock

**Affiliations:** ^1^Imperial College London, Medicine, London, United Kingdom; ^2^Department of Microbiology and Immunology, Peter Doherty Institute for Infection and Immunity, University of Melbourne, Melbourne, VIC, Australia; ^3^ARC Centre of Excellence in Convergent Bio-Nano Science and Technology, University of Melbourne, Melbourne, VIC, Australia; ^4^Melbourne Sexual Health Centre, Department of Infectious Diseases, Alfred Health, Central Clinical School, Monash University, Melbourne, VIC, Australia; ^5^IAVI Human Immunology Laboratory, Imperial College London, London, United Kingdom

**Keywords:** allotype, G1m3, G1m1, HIV, vaccines

An author name was incorrectly spelled as **Jill Gilmore**. The correct spelling is **Jill Gilmour**. The authors apologize for this error and state that this does not change the scientific conclusions of the article in any way.

The original article has been updated.

## Conflict of Interest Statement

The authors declare that the research was conducted in the absence of any commercial or financial relationships that could be construed as a potential conflict of interest.

