# Supplementary material for: Immunoglobulin G1 Allotype Influences Antibody Subclass Distribution in Response to HIV gp140 Vaccination
Source: Front Immunol. 2017 Dec 20;8:1883. doi: 10.3389/fimmu.2017.01883 (PMC5742328; doi:10.3389/fimmu.2017.01883)
Supplement: Supplementary file 1 [file Data_Sheet_1.PDF]

Table S1. Primer set for primary G1m1 PCR.

Allotype-specificity	Primer	Primer Sequence	Length
**G1m1**	Forward (5’ to 3’)	GCAACACCAAGGTGGACAAGAA	22 bp
	Reverse (5’ to 3’)	ACCTGGTTCTTGGTCAGCTCA	21 bp

Table S2. Primer set for primary G1m3-PCR.

Allotype-specificity	Primer	Primer Sequence	Length
**G1m3**	Forward (5’ to 3’)	GCAACACCAAGGTGGACAAGAG	22 bp
	Reverse (5’ to 3’)	ACCTGGTTCTTGGTCATCTCC	21 bp

Table S3. Primer set for secondary G1m3-PCR.

Allotype-specificity	Primer	Primer Sequence	Length
**G1m3**	Forward (5’ to 3’)	CCAAATCTTGTGACAAAACTCACACA	26 bp
	Reverse (5’ to 3’)	TTCTCGGGGCTGCCCTTTGGC	21 bp

Table S4. Reagents and program for primary IgG1-allotyping PCR.

Reagent	Volume		PRIMARY PCR			
10x PCR Buffer, Minus Mg	5.0 µl			Temp.	Time	Cycle
10 mM dNTP mixture	1.0 µl		Initialising step	95°C	10 min	1x
50 mM MgCl2	1.5 µl		Denaturation step	94°C	30 sec	G1m1,17: 30x<br>G1m1,3: 20x
Forward (10 uM each)	1.0 µl		Annealing step	60°C	30 sec	
Reverse (10 uM each)	1.0 µl		Extension step	72°C	40 sec (rate: 1 min/kb)	
Template DNA (cDNA)	2.0 µl		Final extension step	72°C	10 min	1x
Taq Polymerase	0.4 µl		Hold	4°C	Cont.	Cont.
dH20	38.1 µl					

Table S5. Reagents and program for secondary IgG1-allotyping PCR.

Reagent	Volume		SECONDARY PCR			
10x PCR Buffer, Minus Mg	5.0 µl			Temp.	Time	Cycle
10 mM dNTP mixture	1.0 µl		Initialising step	95°C	10 min	1x
50 mM MgCl2	1.5 µl		Denaturation step	94°C	30 sec	25x
Forward (10 uM each)	1.0 µl		Annealing step	65°C	30 sec	
Reverse (10 uM each)	1.0 µl		Extension step	72°C	35 sec (rate: 1 min/kb)	
Template DNA (cDNA)	0.01 µl		Final extension step	72°C	10 min	1x
Taq Polymerase	0.4 µl		Hold	4°C	Cont.	Cont.
dH20	40.1 µl					

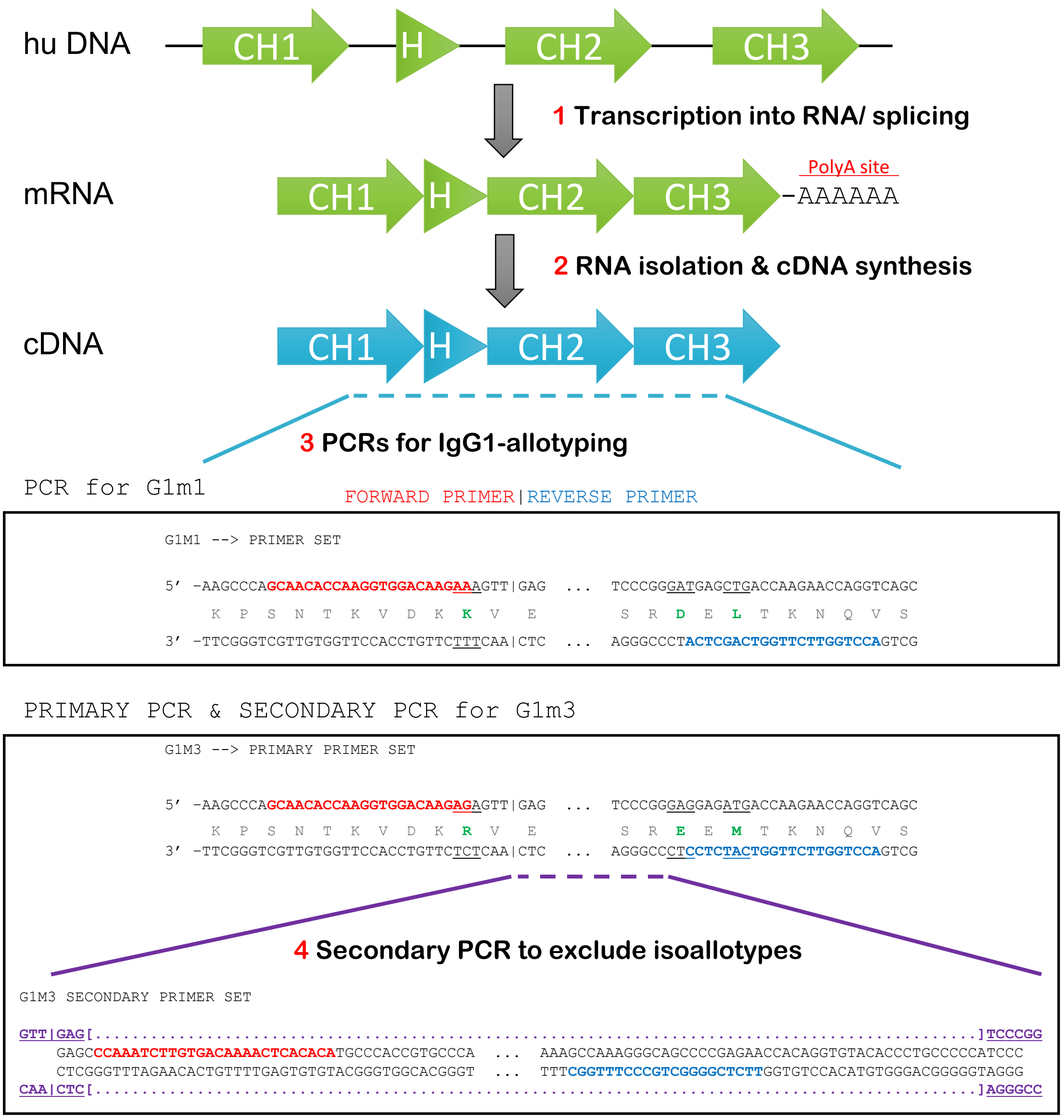

Fig S1. Schematic workflow for determination of IgG1-allotypes G1m3 and G1m1 via PCR.

Uncropped gel:

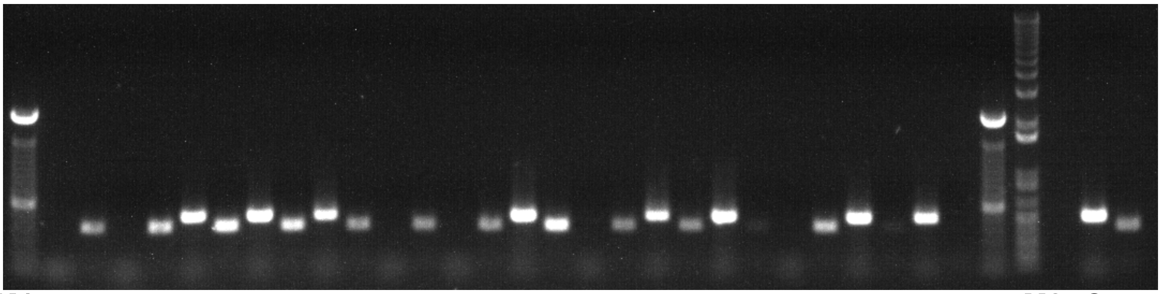

M1 M1 M2 Ctrl.

Fig S2. Complete gel picture used to prepare main text figure 1.

Agarose gel showing all IgG1-allotype PCR products from the X001 study (Participants 001 to 014). Band sizes are 400 bp for G1m3 and 463 bp for G1m1. Molecular weight markers used are the Track-It 100bp ladder (M1) and the Track-It 1 Kb Plus ladder (M2). PCR products from samples of a known homozygous allotype are shown as PCR controls at the right of the gel.

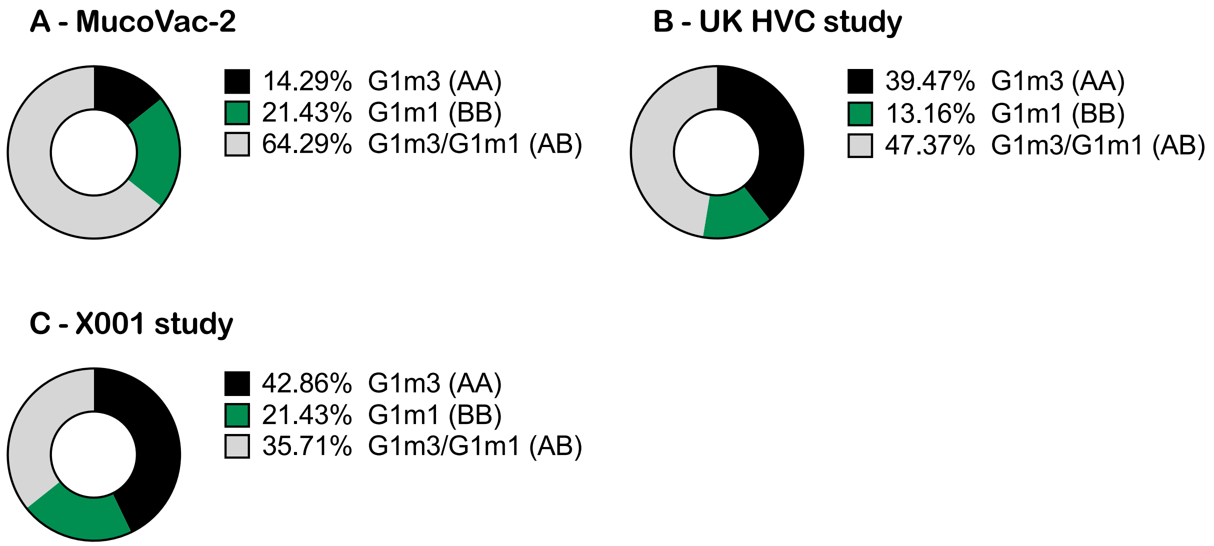

Fig S3. Distribution of IgG1-allotypes in G1m3 and/or G1m1 in cohorts from three previously published HIV vaccine studies.

A - MUCOVAC2, n=20 (Cosgrove et al., 2016), B - UK-HVC Spoke 003, n=38 (Joseph et al., 2017) and C- X001, n=14 (Kratochvil et al., 2017). Due to the limited availability of samples, IgG1-allotypes G1m3 and G1m1 were exclusively determined serologically via ELISA for MUCOVAC2 and the UK HVC Spoke 003 study.

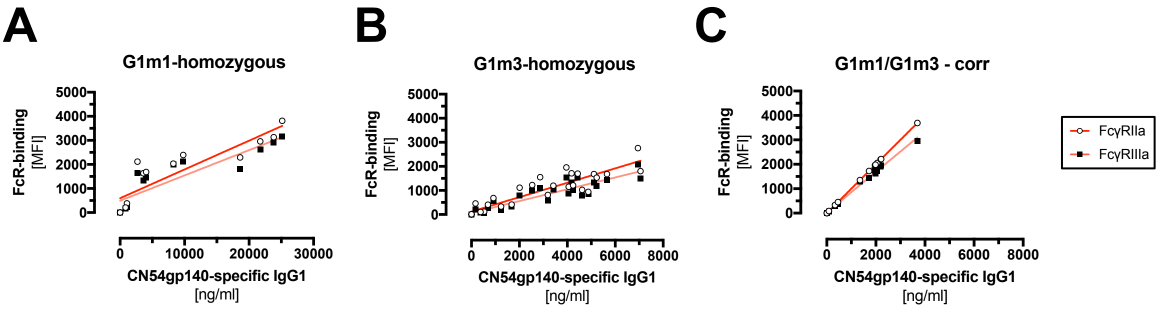

r= 0.969 (p=****)	r= 0.886 (p=****)	r= 1 (p=****)	(IgG1 vs FcγRIIa)
r= 0.973 (p=****)	r= 0.905 (p=****)	r= 0.987 (p=****)	(IgG1 vs FcγRIII)

Fig S4. HIV-specific IgG1 levels (x-axis correlate) with Fc-receptor binding X001 study participants.

(A) homozygous G1m1-carriers, (B) homozygous G1m3-carriers and (C) individuals heterozygous for G1m1/ G1m3. Below the graph Spearman r-values are shown (****p<0.0001). MFI – Median fluorescence intensity.
